# Predictors of chronic cerebrospinal venous insufficiency procedure use among older people with multiple sclerosis: a national case–control study

**DOI:** 10.1186/s12913-015-0835-y

**Published:** 2015-04-16

**Authors:** Michelle Ploughman, Olivia J Manning, Serge Beaulieu, Chelsea Harris, Stephen H Hogan, Nancy Mayo, John D Fisk, A Dessa Sadovnick, Paul O’Connor, Sarah A Morrow, Luanne M Metz, Penelope Smyth, Penelope W Allderdice, Susan Scott, Ruth Ann Marrie, Mark Stefanelli, Marshall Godwin

**Affiliations:** Recovery and Performance Laboratory, Rehabilitation Research Unit, Faculty of Medicine, Memorial University, Rm 400, L.A. Miller Centre, 100 Forest Rd, St. John’s, NL Canada; Eastern Health Authority, St. John’s, NL Canada; Clinical Epidemiology, McGill University, Montreal, QC Canada; Department of Psychiatry, Faculty of Medicine, Dalhousie University, Halifax, NS Canada; Department of Medical Genetics and Division of Neurology, Faculty of Medicine, University of British Columbia, Vancouver, BC Canada; Department of Neurology, St. Michaels Regional Hospital, Toronto, ON Canada; London Health Science Centre, London, ON Canada; Clinical Neurosciences, University of Calgary, Calgary, AB Canada; Department of Neurology, University of Alberta, Edmonton, AB Canada; Departments of Internal Medicine and Community Health Sciences, University of Manitoba, Winnipeg, MB Canada; Department of Neurology, Faculty of Medicine, Memorial University, St. John’s, NL Canada; Primary Health Care Research Unit, Faculty of Medicine, Memorial University, St. John’s, NL Canada

**Keywords:** Multiple sclerosis, Chronic cerebrospinal venous insufficiency, CCSVI, Liberation, Zamboni, Alternative therapies, Controversy, Patient autonomy, Health decision-making

## Abstract

**Background:**

Following the initial reports of Chronic Cerebrospinal Venous Insufficiency (CCSVI) and the purported curative potential of venoplasty, (coined the ‘liberation’ procedure) Canadians living with multiple sclerosis (MS) began to travel abroad to receive the unregulated procedure, often placing them at odds with their health providers. The purpose of this study was to determine the factors influencing older MS patients’ decision to undergo the procedure in order to develop more specific and targeted health information.

**Methods:**

We performed secondary analysis of data collected as part of the ‘Canadian Survey of Health Lifestyle and Aging with MS’ from people over the age of 55 years with MS symptoms for 20 or more years. The survey consisted of self-reported information on impairments, disability, participation, demographics, personal and environmental factors. In order to compare respondents who underwent the procedure to those who did not and to develop a predictive model, we created a comparison group using a case–control algorithm, controlling for age, gender and education, and matching procedure cases to controls 1:3. We used multivariate stepwise least likelihood regression of ‘a priori’ variables to determine predictive factors.

**Results:**

The prevalence of the ‘liberation’ procedure in our sample was 12.8% (95/743), substantially lower than reported in previous studies of complementary/alternative treatments in MS. The predictive model contained five factors; living alone (Odds ratio 0.24, 95%CI 0.09-0.63), diagnosis of anxiety (Odds ratio 0.29, 95%CI 0.10 - 0.84), rating of neurologist’s helpfulness (Odds ratio 0.56, 95%CI 0.44 -0 .71), Body Mass Index (Odds ratio 0.93, 95%CI, 0.89 - 0.98) and perceived physical impact of MS (Odds ratio 1.02, 95%CI 1.01 - 1.04).

**Conclusions:**

Predictive factors differed from previous studies of complementary/alternative treatment use likely due to both the invasiveness of the procedure and the advanced age of our study cohort. Our findings suggest that health professionals should target information on the risks and benefits of unregulated procedures to those patients who feel dissatisfied with their neurologist and they should include family members in discussions since they may be providing the logistical support to travel abroad and undergo the ‘liberation’ procedure. Our findings may be applicable to others with chronic disabling conditions who contemplate the user-pay unregulated invasive procedures available to them.

**Electronic supplementary material:**

The online version of this article (doi:10.1186/s12913-015-0835-y) contains supplementary material, which is available to authorized users.

## Background

Diagnosed in young and middle-aged adults during their career and family-building years, multiple sclerosis profoundly impacts health-related quality of life [1-3]. In 80% of cases, MS is characterized by an unpredictable relapsing-remitting course, affecting movement, balance, sensation and vision [4]. Twenty five years after disease onset, 90% of those with MS will have significant functional limitation but the long term consequences of the disease are highly variable among individuals [5,6].

Recognized as an idiopathic autoimmune degenerative disease, the specific etiology and pathophysiology of MS is still under debate [7]. The theory of Chronic Cerebrospinal Venous Insufficiency (CCSVI) emerged in 2009 which purported that impaired venous drainage in the jugular and azygous veins created reflux of blood in the deep cerebral veins which subsequently induced MS inflammatory lesions [8]. In some regions of the world, user-pay clinics began to provide the endovascular treatments (venoplasty) to open obstructed areas in the veins which was coined the ‘liberation’ or the ‘Zamboni’ procedure by some [9]. Recent results of observational and randomized controlled trials [10,11] suggest that CCSVI is a normal incidental finding that is not related to MS lesions.

Canada has the fifth highest rate of MS in the world [12] and when Canadian television media [13] reported the ‘liberation’ procedure as a cure for MS, the response from the MS community was unprecedented [9]. Despite the invasive nature of the surgery and the associated risks, it is believed that thousands of Canadians living with MS began travelling abroad to undergo the procedure. Health professionals and MS patients found themselves often on opposite sides of the debate [14]. Three recent studies, using qualitative methods, have reported that Canadians living with MS decided to receive the procedure because they lost faith in the Canadian health care system [15,16]; they were encouraged by others who had received it [15,16], and the procedure gave them hope [17,16]. Quantitative predictors of CCSVI procedure use have not been described. There may be some parallels between predictors of CCSVI procedure and those of complementary and alternative medicine (CAM). Previous studies have shown that CAM users are typically highly educated, middle-aged women [18,19]. They also tend to report a low health-related quality of life, declare dissatisfaction with their health care professionals, and express a desire to have more control over treatment decisions [20-22]. However, following one attempt to create a predictive model of CAM use in MS using a large national registry, the authors suggested that a better model fit could be achieved by including disease-related variables and personal factors [18].

We undertook this study to determine the factors propelling the decision to travel abroad and obtain the ‘liberation’ or CCSVI procedure among Canadians; critical knowledge that could potentially assist health care professionals in targeting the right information to vulnerable patients. To determine the factors associated with undergoing the ‘liberation’ procedure, we analysed data from the ‘Canadian Survey of Health Lifestyle and Aging with MS’ collected between May 2010 and December 2012 [23], during the height of the ‘liberation’ procedure controversy.

## Methods

### Survey design

We performed secondary analysis of cross-sectional data collected as part of the ‘Canadian Survey of Health, Lifestyle and Aging with MS’ which was approved by Research Ethics Boards in eight Canadian provinces (refer to Additional file 1). Details of the survey and sampling are described elsewhere [20]. This purposeful sample included older Canadians, over the age of 55 and had MS symptoms for 20 or more years, who had been recruited through MS clinics, MS Society chapters and newspapers. The survey, constructed based on the World Health Organization Framework [24], consisted of patient-reported validated outcome tools and custom-designed questions measuring impairments (eg. Hospital Anxiety and Depression Scale [25]), disability (eg. Barthel Index [26]), participation (eg. Frenchay Activities Index [27]), health-related quality of life (eg. Multiple Sclerosis Impact Scale [28]), and personal (eg. Personal Resource Questionnaire-2000 [29], and Simple Lifestyle Indicator Questionnaire [30]) and environmental factors (eg. finances, health care). One survey question asked participants to list “supplements or alternative treatments for your MS now or in the past (anything not given to you through a doctor’s prescription such as liberation treatments, bee sting therapy or herbal supplements)”. They were then asked to rate the helpfulness of the therapy on a scale from 1-not at all helpful to 5- extremely helpful. They were also asked to list and rate the helpfulness of health care professionals they have worked with. Additionally, two open-ended questions at the end of the survey asked respondents “From your point of view, what are the most important things that help you live long and healthy with MS” and “If you would like to make any final comments about this questionnaire or the study itself, please record them in the space provided below”.

### Participants

We used two methods to identify respondents in the survey database who underwent the ‘liberation’ procedure. The first was by the response to the question about use of alternative therapies (which included the ‘liberation’ procedure) and the second, by performing a key word search (‘liberation’, ‘CCSVI’, ‘Zamboni’ and ‘chronic cerebrospinal venous insufficiency’) of all response data.

In order to compare respondents who underwent the procedure to those who did not and to develop a predictive model, we created a comparison group using a case–control algorithm. Each ‘procedure’ case was matched to three controls (‘no procedure’) based on gender, age (±3 years), and total years of formal education (±3 years). The differences between the group means of the case matching variables (age, gender, and education) and their ability to predict the ‘liberation’ procedure were first confirmed to be insignificant using ANOVA and simple linear regression respectively. After determining minimum and maximum acceptable limits for education and age for each ‘procedure’ case, we created a Microsoft Excel pivot table identifying all possible controls for each case. An equation was applied to select three controls at random. These cases and controls were moved into their own database (IBM SPSS v20) for analysis.

### Data analysis

#### Quantitative

The characteristics of cases and controls were compared using one-way ANOVA or in the case of binary variables, chi-square. Since we aimed to identify factors which influenced whether to undergo the procedure or not, we identified ‘a priori’ explanatory variables based on previous research [18,17,15] in five categories; (1) satisfaction with conventional healthcare, (2) mental health, (3) MS severity, (4) resources (personal and financial), and (5) lifestyle/overall health (Table 1). In the first step of building an explanatory model, each ‘a priori’ variable (independent variable) was separately entered into a simple binary logistic regression with the dependent variable, ‘liberation’ procedure (yes/no). In the second step, only those variables that significantly predicted ‘procedure’ or ‘no procedure’ (p < 0.05) were entered into forward and backward stepwise likelihood ratio multivariate regression. In both steps, colinearity between variables was checked. We used the Hosmer and Lemeshow Test to assess model fit in which a non-significant p value indicates a good fit. Analysis was performed in SPSS v20 with significance set at p < 0.05.

**Table 1:** **Potential explanatory variables**

**Category**	**Variable**	**Category**	**Variable**
**Satisfaction with conventional health care**	Number of alternative therapies (not liberation)	**MS severity**	Participation (FAI domestic)
	Number of medications		Participation (FAI leisure)
	General practitioner rating		Participation (FAI outdoor)
	MS nurse rating		Physical impact of MS (MSIS-29)
	Physiotherapy and occupational therapy rating		Psychological impact of MS (MSIS-29)
	Neurologist helpfulness rating		Years with MS
**Mental health**	Diagnosed anxiety		Disability (Barthel Index)
	Diagnosed depression	**Lifestyle/Overall health**	Number of co-morbid conditions
	Anxiety symptoms (HADS-A)		Health habits (SLIQ)
	Depression symptoms (HADS-D)		Body Mass Index (BMI)
	Resilience (RS)	**Resources**	Social support (PRQ-2000)
	Fatigue		Living situation
			Financial resources

HADS-A or -D, Hospital Anxiety and Depression Scale –Anxiety or -Depression; RS, Resilience Scale; FAI, Frenchay Activities Index; SLIQ, Simple Lifestyle Indicator Questionnaire; BMI, Body Mass Index; PRQ-2000, Personal Resource Questionnaire version2000.

#### Qualitative

Since the database included answers to open-ended questions about living with MS as well as unsolicited comments, advice and stories from participants on many subjects, we used key word searches of these data to identify potential text related to the ‘liberation’ procedure. Key words included ‘liberation’, ‘CCSVI’, ‘Zamboni’, ‘neurologist’, ‘health’, ‘medical’, ‘team’, ‘care’, ‘cure’, ‘treatment’, ‘procedure’, ‘profession’, ‘trust’, ‘government’, and ‘alternative’. The text was reviewed to ensure that the discussion related to the ‘liberation’ procedure and then divided by respondent group, ‘procedure’ or ‘no procedure’. Two of the researchers (MP and OJM) independently read the text multiple times to extract the essential elements. We used a thematic content analysis approach to identify key themes in the respondents’ accounts [31]. The coders each created an initial coding grid, then by consensus collapsed or deleted redundant codes to create a final thematic scheme. One researcher (OJM) then coded all text according to the scheme and gathered quotes that embodied the essence of the theme. Both researchers then collaboratively described the key messages and chose quotes that represented those messages.

## Results

### Quantitative findings

The 743 survey respondents ranged in age from 55 to 88 years (64.6 ± 6.18) and reported having MS symptoms for 32.9 (±9.5) years. The number of women outnumbered men 3.48:1. Ninety percent of respondents were either retired or unemployed and 28% reported that they were no longer able to walk or were bedridden. Respondents had on average 1.5 years of post-secondary education and 85.2% of respondents reported having at least one comorbid condition. Seven hundred and nine people completed the open-ended questions (95%). We identified 95 people in the database who reported that they had received the ‘liberation’ procedure (12.8% of the entire sample). Ninety-one participants indicated they had the ‘liberation’ procedure in the alternative therapies section of the survey and four participants disclosed they had the procedure in the final section of the survey open for comments. Eighty-seven respondents rated the helpfulness of the ‘liberation’ procedure (1 = least helpful, 5 = most helpful). Forty-nine (56%) described it as helpful while the remainder were neutral or found it not helpful.

We successfully matched controls to cases 3:1 and created a new database of cases and controls (n = 380). The characteristics of the ‘procedure’ group and the ‘no procedure’ group are outlined in Table 2 with no significant differences in the matched variables using ANOVA and no significant relationship of matched variables and obtaining the ‘liberation’ procedure.

**Table 2:** Participant Characteristics

**Characteristic**	**Procedure (n = 95)**	**No Procedure (n = 285)**
Sex	77 F/18 M	224 F/61 M
Age (yrs)	63.25 ± 5.63	63.69 ± 5.49
Total Education (yrs)	13.52 ± 2.52	13.32 ± 2.16

Following simple binary logistic regression analysis of the ‘a priori’ explanatory factors, seven were significantly predictive of receiving the ‘liberation’ procedure. Odds ratios (EXP β) and 95% confidence intervals are indicated in Table 3. The ‘procedure’ group provided significantly lower ratings of their neurologists’ helpfulness (but not significantly lower ratings of other health professionals). They were also healthier with fewer co-morbid conditions, lower reported body mass index and were less likely to be diagnosed with anxiety; all these factors were predictive of group assignment. Although we found no difference in disability measured by the Barthel Index (no procedure 78.18 ± 1.42; procedure 73.50 ± 25.25), the ‘procedure’ group reported higher perception of the impact of their MS and less participation in household activities of daily living (ADL) measured by the FAI. Social support or financial situation were not predictive of receiving the ‘liberation’ procedure, however we did find that living situation was predictive, in that those who were living alone were significantly less likely to have the procedure.

**Table 3:** **Significant explanatory variables for ‘liberation’ procedure**

**Category**	**Variable**	**Procedure**	**No Procedure**	**EXP ß**
		Mean ± SD	Mean ± SD	(95% CI))
**Satisfaction with Conventional Health Care**	Neurologist Helpfulness Rating (rated 1–5)	2.93 ± 1.46*	3.94 ± 1.09	0.54*
				(0.43-0.66)
**Mental Health**	Diagnosed Anxiety	5/95 (5.26%)**	45/285 (15.79%)	0.30***
				(0.11-0.77)
**MS** **Severity**	Physical Impact of MS (MSIS-29)	64.46 ± 19.06*	55.32 ± 20.20	1.02*
				(1.01-1.04)
	Participation (FAI Domestic Activities)	12.88 ± 5.20***	14.45 ± 5.22	0.95***
				(0.91-0.99)
**Lifestyle/Overall Health**	Number of Co-morbid conditions	1.92 ± 1.76**	2.61 ± 2.12	0.83**
				(0.73-0.95)
	Body Mass Index (BMI)	23.81 ± 4.66**	25.79 ± 6.07	0.94**
				(0.91-0.98)
**Resources**	Living Situation “I live alone”	8/95 (8.42%)*	68/285 (23.86%)	0.293**
				(0.14-0.64)

*p < 0.001 **p < 0.01, ***p < 0.05.

Before building a predictive model with the minimum number of explanatory factors, we examined the correlations between the factors (Table 4). As expected, there were minimal to moderate correlations between related variables (bolded in Table 4). Moderate correlations over 0.30 existed (and were expected) between physical impact of MS and participation, between number of co-morbid conditions and BMI and between number of co-morbid conditions and anxiety diagnosis. We conducted the model fit analysis adding and deleting these correlated variables until we achieved the best fit. There was no significant effect of colinearity.

**Table 4:** **Correlation between explanatory variables**

**Variable**	**Neurologist rating**	**Diagnosed anxiety**	**Physical Impact of MS**	**Participation (FAI Domestic)**	**Number of Co-morbid conditions**	**BMI**
Neurologist Helpfulness Rating	1					
Diagnosed Anxiety	−0.015	1				
Physical Impact of MS	−0.268**	0.070	1			
Participation (FAI Domestic)	0.203**	−0.052	−0.575*	1		
Number of Co-morbid conditions	0.012	0.349*	0.033	−0.003	1	
BMI	−0.029	0.076	0.041	−0.014	0.282*	1
Living Situation “I live alone”	0.005	−0.019	0.023	−0.001	0.010	−0.003

*p < 0.001, ** p < 0.01, ***p < 0.05.

All significant explanatory variables (Table 3) were entered into forward and backward stepwise likelihood ratio regression in order from most to least predictive (β). The Hosmer and Lemeshow Test showed that the best model fit included five variables (Table 5: chi square 9.94, p = 0.267) with 77.9% overall correct prediction (32.9% correct procedure group, and 92.7% no procedure group); only 8 cases misclassified as ‘no procedure’.

**Table 5:** **Final model predicting the ‘liberation’ procedure among older Canadians with MS**

**MODEL COMPONENTS**	**ß**	**EXP ß**
		**(95% CI)**
**1. Living situation (living alone)**	−1.41	0.24
		(0.09-0.63)
**2. Diagnosed anxiety**	−1.23	0.29**
		(0.10 - 0.84)
**3. Neurologist helpfulness rating**	−0.58	0.56*
		(0.44 - 0.71)
**4. Body Mass Index**	−0.07	0.93***
		(0.89 - 0.98)
**5. MSIS Physical Impact Scale**	0.02	1.02***
		(1.01 - 1.04)

*p < 0.001, ** p < 0.01, ***p < 0.05.

The model consisted of living situation (living alone), diagnosis of anxiety, rating of neurologists’ helpfulness, BMI and the score on the MSIS-29 Physical Impact Scale. When participants were living alone, they were 76% less likely to have the procedure (EXP β 0.24). Those diagnosed with anxiety (but not depression or other comorbid conditions) were 71% less likely to have the ‘liberation’ procedure (EXP β 0.29). When participants rated their neurologist’s helpfulness as high, they were 44% less likely to have the procedure (EXP β 0.56). The two remaining factors (BMI and MS Physical Impact) had much lower, although significant, predictive value. Those respondents with higher reported BMI were 7% less likely to have the procedure (EXP β 0.93) and finally, if participants had a high MSIS-29 Physical Impact Score, they were about 8% more likely to have the procedure (EXP β 1.02). To ensure these findings were correct, we performed the same analysis on the full dataset (n = 743) with similar results (data not shown).

### Qualitative findings

Using the key word search, we found 61 text strings discussing the ‘liberation’ procedure; 34 ‘procedure’ group and 27 ‘no procedure’ group. The main themes and their frequencies are outlined in Table 6.

**Table 6:** **Frequency of themes by group**

**TOPIC**	**Procedure**	**No procedure**
**Canadian healthcare, trust and credibility**	16	16
**Hope for a cure**	5	2
**Perceived outcome of procedure**	Positive = 11	0
	Negative = 2	
**Desire for the procedure**	0	9

#### Canadian healthcare, trust and credibility

The ‘procedure’ and ‘no procedure’ groups described polar opinions about their trust in the Canadian healthcare system. Members of the ‘no procedure’ group described how they were fortunate to have the support of their neurologists and family doctors; “good medical support is essential”. Those who described a trusting relationship also commented how this trusted source was their primary source of health information; “I trust the MS Society’s [information]”. One respondent in the ‘no procedure’ group described how she knew better than “to surf on the computer” and was “skeptical about new therapies”.

Participants who had the ‘liberation’ procedure expressed frustration towards the government and health providers regarding the availability of the procedure in Canada (n = 10). Only one participant in the ‘no procedure’ group expressed these concerns. Several respondents from the ‘procedure’ group described how they were willing to try new therapies but felt that physicians and the MS Society were “too slow; too controlled by the pharmacy companies and too short-sighted”. Those returning to Canada after receiving the procedure abroad felt that they were “on [their] own” and “let down by my provincial healthcare”.

#### Hope for a cure

Participants uniformly described how they are still “hoping for a cure” and one respondent added “so I get my life back”. One participant in the ‘procedure’ group stated “I don’t give up and I always think that there will be a cure for MS in the future so I do the best I can until then.” For respondents in both groups, the promise of a cure touted by the ‘liberation’ procedure information was encouraging.

#### Perceived outcome of the procedure

Eleven participants in the ‘procedure’ group described improvements in balance, fatigue and pain following the ‘liberation’ procedure. Several qualified their endorsement by explaining that they were not entirely free of MS symptoms as a result of the procedure. For example, one respondent stated, “I used to see through a cloud and now that cloud is gone. I feel that this was well worth it even if I still have balance and fatigue problems. They will go away when the attack halts”. The spouse of one ‘procedure’ group participant felt her husband had initial improvements in “balance, energy, improved walking, warm hands and feet and speech. These lasted for six months but he has regressed since then.”

#### Desire for the procedure

Nine participants in the ‘no procedure’ group described a desire to undergo the ‘liberation’ procedure. Several stated that they had “nothing to lose” but they “do not have the money to go.” They described feeling frustrated while waiting until the procedure is made available in Canada. One participant said, “Right now, I don’t see any light at the end of the tunnel because Canadian physicians and surgeons are not willing to support the ‘liberation’ treatment that has had positive results in other countries.”

## Discussion

Although user-pay invasive, unregulated procedures are available to those who seek them, this study is the first to describe the prevalence and predictors of one such procedure, the ‘liberation’ procedure among people with MS. The purpose of this study was to understand why older MS patients chose to have the ‘liberation’ procedure in order to target and tailor health information for those patients who may be more likely to undertake such interventions. We used a mixed methods approach to secondary data analysis from Canadian national survey data. Using an age, gender and education-matched, case–control algorithm, we created a 3:1 sample of ‘no procedure’ and ‘procedure’. Both groups (with and without the procedure) were on average about 63 years old with 1.5 years of post-secondary education, with women outnumbering men, typical of MS demographics in general (3.48:1 full database; 3.67:1 ‘no procedure’: 4.27:1 ‘procedure’). Our findings may be applicable to others with chronic disabling conditions since the factors that influenced decision-making among this MS cohort may be similar to those considering surgical procedures that are purported as the cure for spinal cord injury and cancer, among others.

The prevalence of the ‘liberation’ procedure in our sample was 12.8%, a quarter of recent reported CAM use in Nordic countries among people with MS [32] and less than half of the 30% current use of alternative therapies reported by Marrie [18] among the North American Research Consortium on Multiple Sclerosis (NARCOMS) sample of 20,778 people with MS in North America. Although there are other invasive procedures available for purchase, such as unregulated stem cell transplants and ‘reconstruction’, advertised to treat conditions such as cancer and spinal cord injury, the prevalence of these have not been reported making it difficult to compare our results to other cohorts. Clearly, use of CAM such as evening primrose oil, vitamins or visiting a nutritionist or chiropractor is accessible and low risk thus it not surprising that CAM use is much greater than an alternative surgical procedure such the ‘liberation’ procedure.

In order to create a case–control database to develop a predictive model of ‘liberation’ procedure, we first determined that age, gender and educational level were not different or significantly predictive of receiving the ‘liberation’ procedure. We found that living alone, diagnosis of anxiety, higher rating of neurologist’s helpfulness, higher BMI and lower perceived physical impact of MS reduced the respondent’s likelihood of undergoing the procedure.

Our findings differ from previous research on CAM use in MS. For example, MS patients who utilize unconventional/CAM therapies are typically college educated [18,19,32,33] middle aged females [18,19,32] with higher than average income [32,34]. We found no predictive ability of these previously reported demographic factors (age, gender, education, finances) in our model. Considering the cost of the procedure, we were surprised that financial situation was not predictive, however our previous qualitative findings [15] suggest that some people overcame financial barriers by fundraising in their communities. Gender was also not predictive and we found that men were equally likely to undergo the ‘liberation’ procedure as women. These discrepancies may be related to the fact that we sampled an older cohort and that factors predicting CAM use may have little overlap with factors predicting a surgical procedure such as the ‘liberation’ procedure.

Living alone was a novel predictor that has not been described in CAM research thus far. Since we found that neither personal resources (PRQ-2000) nor financial resources were predictive, we think, based on our previous qualitative findings [15,17] that those who live with others have more practical support in the home to investigate, plan and accompany the person with MS to travel abroad for the procedure. We previously reported that in some cases, people with MS felt strongly influenced by the opinions of family and friends, however in the current study, respondents indicated that they were quite agreeable and in fact enthusiastic about the procedure despite varying benefits. Qualitative findings from this study concur with others suggesting that the procedure fostered hope [16,17]. This suggests that the role of the family should be taken into consideration when providing patient education and initiating a discourse about the risks and benefits of unregulated procedures, as family members may influence health decision-making at least in this older cohort of people with MS.

It was interesting that a diagnosis of anxiety (but not anxiety symptoms measured by HADS-A) was associated with less likelihood of receiving the procedure. Anxiety was included in the full list of co-morbid conditions and both factors (anxiety and comorbidity) were initially predictive of ‘liberation’ using simple regression. On further examination of data and model fit, we found that the predictive ability of ‘number of comorbid conditions’ was actually the contribution of anxiety diagnosis and not other health conditions such as heart disease or arthritis. We are the first to report such a finding. We postulate that people with an anxiety diagnoses were less likely to attempt the organization, travel and risks required to undergo the procedure. Our qualitative findings support that some people desired the procedure even though they did not receive it.

People who reported lower BMI were significantly more likely to have the procedure. According to Centers for Disease Control and Prevention [35], the ‘no procedure’ group with a mean BMI of 25.8 would be classified as overweight while the ‘procedure’ group at 23.8 would be in the normal range. Since we found that BMI was positively correlated with number of comorbid conditions, perhaps respondents with a lower BMI may have fewer health risks to consider when making decisions whether to undergo the ‘liberation’ procedure. Our findings differ from those reported by Schwartz and group that suggested that people with MS with at least one comorbid condition were more likely to use unconventional therapies [34]. This difference may be related to the degree of risk associated with typical unconventional therapies (supplements, diets etc.) compared to the ‘liberation’ procedure.

The predictive ability of rating of neurologist’s helpfulness was not surprising since our qualitative findings suggested that neurologists were a target of frustration for patients who wished to have the procedure in Canada and could not. Snyder et al. [16] and Ploughman et al. [15] confirmed that people who decided to undergo the procedure were frustrated with the Canadian health system. Others have reported that higher health care provider ratings were either predictive of CAM use [33] or had no effect [34]. When Campbell and colleagues [33] investigated the predictive effect of health care provider ratings on CAM use, they found that satisfaction with care was not predictive. However, their respondents rated their overall satisfaction with all health care providers rather than specific professionals as in our study. We also found modest but significant correlation between rating of neurologist helpfulness and the physical impact of MS and household participation (Table 4), suggesting that people with higher impact of MS (but not level of disability measured by Barthel) feel greater dissatisfaction with neurologists (but not family doctors, nurses or therapists).

In terms of health and MS severity, CAM users tend to have lower self-reported physical health [18,19] or more progressive disease [33]. MS patients who are CAM users are also more likely to utilize more conventional healthcare [18,34]. Although disability, measured by the Barthel Index was not predictive in this study, the perceived physical impact of MS measured by the MSIS-29 was predictive. The inconsistencies between findings may be attributed to the differences between CAM and invasive procedures or the difference between the constructs of reported disability, perceived impact of MS, perceived physical health and perceived overall health. There may be a disconnect between disability and perceived health among this older cohort who have lived with MS for more than 20 years as reported by others [2,3].

Although we used a mixed-methods approach in a national survey to gain an understanding of factors influencing the decisions surrounding the ‘liberation’ procedure, there are some limitations to the study. First of all, we collected data at one point in time so we are unable to make causal inferences. We performed secondary analysis on quantitative and qualitative data that was collected for another use so we may have seen different results in a survey designed specifically to investigate CCSVI. Furthermore since we did not ask participants the date of their ‘liberation’ procedure, we do not know the temporal relationship between having the procedure and completing the survey. Opinions and beliefs about the ‘liberation’ procedure could change over time. We also surveyed older people with MS over 55 years of age with MS symptoms for more than 20 years, making our findings difficult to translate to a younger cohort. Furthermore, it is likely that those who live in long term care facilities and/or did not attend MS clinics may be underrepresented.

## Conclusions

To determine the factors associated with undergoing the ‘liberation’ procedure, we performed secondary analysis of survey data collected during the height of the ‘liberation’ procedure controversy in Canada. The prevalence of the ‘liberation’ procedure in our sample was 12.8%, substantially less than reported in studies of other CAM or unconventional treatments in MS. The predictive model contained five factors; living alone, diagnosis of anxiety, rating of neurologist’s helpfulness, BMI and perceived physical impact of MS. Predictive factors differed from previous studies of CAM use likely due to both the invasiveness of the procedure and the advanced age of our study cohort. Our findings suggest that health professionals should target information on the risks and benefits of unregulated procedures to those patients who feel dissatisfied with their neurologist and they should include family members in discussions since they may be providing the logistical support to travel abroad and undergo the ‘liberation’ procedure. Our findings may be applicable to others with chronic disabling conditions who contemplate the user-pay unregulated invasive procedures available to them.

## Additional file

Additional file 1:
**Health Lifestyle and Aging with MS Study Ethics boards.**

## Abbreviations

BMI: Body mass index
CAM: Complementary and alternative medicine
CCSVI: Chronic cerebrospinal venous insufficiency
FAI: Frenchay Activities Index
HADS-A or -D: Hospital anxiety and depression scale –anxiety or -depression
MS: Multiple sclerosis
MSIS-29: Multiple sclerosis impact scale-29
NARCOMS: North american research consortium on multiple sclerosis
PRQ-2000: Personal resource questionnaire version2000
RS: Resilience scale
SLIQ: Simple lifestyle indicator questionnaire
WHO: World health organization
