# Supplementary material for: Predictors of chronic cerebrospinal venous insufficiency procedure use among older people with multiple sclerosis: a national case–control study
Source: BMC Health Serv Res. 2015 Apr 16;15:161. doi: 10.1186/s12913-015-0835-y (PMC4424567; doi:10.1186/s12913-015-0835-y)
Supplement: Additional file 1: — Health Lifestyle and Aging with MS Study Ethics boards. [file 12913_2015_835_MOESM1_ESM.docx]

**Health Lifestyle and Aging with MS Study**

**Ethics boards**

Human Investigation Committee, Memorial University and Patient Research Ethics Committee, Eastern Health Authority, St. John’s NL Canada

Capital Health Research Ethics Board, Halifax NS Canada

McGill University Health Centre and Montreal General Hospital Research Ethics Office, Montreal QC Canada

Health Sciences Research Ethics Board, University of Western Ontario, London ON Canada

Research Ethics Board, St. Michael’s Hospital, Toronto ON Canada

Research Ethics Board. Bannatyne campus, University of Manitoba, Winnipeg MB Canada

Behavioural Research Ethics Board, University of Saskatchewan, Saskatoon SK Canada

Health Research Ethics Board, University of Alberta, Alberta Health Services, Edmonton AB, Canada

Behavioural Research Ethics Board, University of British Columbia, Vancouver BC, Canada
